# The association between residual neuromuscular blockade (RNMB) and critical respiratory events: a prospective cohort study

**DOI:** 10.1186/s13741-021-00183-7

**Published:** 2021-05-04

**Authors:** Faraj K Alenezi, Khalid Alnababtah, Mohammed M Alqahtani, Lafi Olayan, Mohammed Alharbi

**Affiliations:** 1 Anesthesia Technology Program, College of Applied Medical Sciences, King Saud bin Abdul-Aziz University for Health Sciences, Riyadh, Saudi Arabia; 2 King Abdullah International Medical Research Center, Riyadh, Saudi Arabia; 3 School of Nursing and Midwifery, Faculty of Health, Education and Life Sciences, BCU, Birmingham, UK; 4 Respiratory Therapy Program, College of Applied Medical Sciences, King Saud bin Abdul-Aziz University for Health Sciences, Riyadh, Saudi Arabia

**Keywords:** Neuromuscular blocking drugs, Residual neuromuscular blockade, Critical respiratory events

## Abstract

**Background:**

Inadequate neuromuscular recovery might impair pulmonary function among adult patients who undergo general anaesthesia and might thus contribute to critical respiratory events in the post-anaesthesia care unit (PACU). The pilot study aims to understand the baseline incidence of residual neuromuscular blockade (RNMB) and postoperative critical respiratory events (CREs), which are described in a modified Murphy’s criteria in the PACU.

**Method:**

This is a prospective cohort study from January to March 2017 from a tertiary hospital in Saudi Arabia with thirty adult patients over 18 years old scheduled for elective surgery under general anaesthesia with neuromuscular blocking drugs (NMBDs) who were enrolled in the study. The Mann-Whitney *U* tests, chi-square tests and independent-samples *T* tests were used. The train-of-four (TOF) ratios were measured upon arrival in the PACU by using acceleromyography with TOF-Scan. Subjects’ demographics, perioperative data and the occurrence of postoperative CREs in the PACU were recorded.

**Results:**

Twenty-six (86.7%) patients out of thirty in the study have received rocuronium as NMBDs whilst neostigmine as a reversal drug with only 23 (76.7%). The incidence of RNMB (TOF ratio < 0.9) was in 16 patients (53.3%). The incidence of RNMB was significantly higher in female patients (*p* = 0.033), in patients who had not undergone quantitative neuromuscular monitoring before extubation (*p* = 0.046) and in patients with a shorter duration of surgery (*p* = 0.001). Postoperative CREs occurred in twenty patients (66.7%), and there were significantly more of these CREs among patients with RNMB (*p* = 0.001). In addition, a statistically significant difference was observed in the occurrence of CREs according to body mass index (*p* = 0.047).

**Conclusion:**

This research showed that RNMB is a significant contributing factor to the development of critical respiratory events during PACU stay. Therefore, routine quantitative neuromuscular monitoring is recommended to reduce the incidence of RNMB.

## Introduction

Many patients are at high risk of adverse events during the early postoperative period, which might be caused by anaesthetic or surgical procedures. These adverse events include cardiovascular or respiratory complications (Buhre and Rossaint 2003). Therefore, areas known as post-anaesthesia care units (PACU) have been established in many hospitals worldwide, which are intended to provide specialised care and minimise morbidity and mortality through the timely detection or prevention of adverse events (Kiekkas et al. 2014). In clinical practice, neuromuscular blocking drugs (NMBDs) are commonly used by anaesthesiologists during general anaesthesia in order to maintain optimal surgical conditions by maintaining deep muscle relaxation and to facilitate tracheal intubation (Claudius et al. 2009). However, their effects might continue after extubation: this is called residual neuromuscular blockade (RNMB). According to Murphy et al. (2008a), between 33 and 64% of patients have evidence of incomplete neuromuscular recovery or RNMB during their stay in the PACU, despite the use of techniques proven to limit the degree of RNMB, such as reversal drugs. The incidence of RNMB ranges between 26 and 88%, based on the type of NMBDs, reversal drugs or neuromuscular monitoring used (Cammu et al. 2012; Fortier et al. 2015). The RNMB can be reliably detected through the use of neuromuscular monitoring such as train-of-four (TOF) monitoring, which is according to Murphy et al. (2011) is considered one of the most commonly used approaches in clinical practice. This method, however, is used during recovery from the application of NMBDs to objectively decide how well the patient’s muscles are able to function, which includes the application of electrical stimulation to nerves and the recording of muscle responses, called the TOF ratio (Butterworth 2013). The TOF ratio when it is ≥ 0.90 is considered the gold standard for defining adequate neuromuscular recovery, whereas RNMB is present when the TOF ratio < 0.90 (Murphy 2006). Accordingly, The RNMB might place patients at an increased risk of critical respiratory events (CREs) in the PACU. Evidence from observational studies suggests that TOF ratios when it is less than 0.90 are usually associated with the incidence of CREs that include inadequate recovery of pulmonary function, upper airway obstruction, impaired pharyngeal reflexes, decreased muscle coordination, impaired hypoxic ventilatory response and increased risk of aspiration (Cammu et al. 2012; Herbstreit et al. 2009; Murphy et al. 2008a).

To date, the incidence of RNMB and its association with CREs during the PACU stay has not been investigated in Saudi Arabia. In addition, the issue of whether or not neuromuscular monitoring is usually used has not been investigated. Therefore, this pilot study is aiming to understand the baseline incidence of residual neuromuscular blockade (RNMB) and postoperative critical respiratory events (CREs), which are described in a modified Murphy’s criteria, the feasibility of measuring TOF in the PACU stay in a tertiary hospital in Saudi Arabia.

## Methods

### Study design

A pilot prospective cohort design was used in this study which is part of a student project at Cardiff University. The study was conducted in the post-anaesthesia care unit (PACU) at the National Guard Hospital, King Abdulaziz Medical City in Riyadh, Saudi Arabia. The study has been approved by the King Abdullah International Medical Research Centre (KAIMRC) at King Abdulaziz Medical City (IRBC/1235/16). Informed written consent was obtained from all patients before entry into the study. Participants were enrolled between 21st January 2017 and 21st March 2017. All participants were screened for their eligibility based on the inclusion and exclusion criteria (Table 1).

**Table 1:** Inclusion and exclusion criteria

Inclusion	- Patients aged over 18 years - Patients who had undergone elective surgical procedures - Patients who received general anaesthesia only - American Society of Anaesthesiologists (ASA) from 1 to 3
Exclusion	- Patients who were undergoing cardiac, thoracic or neurological surgeries, as well as patients who were undergoing abdominal emergency surgery - Patients who are having a history of pulmonary disease or cardiovascular comorbidities - Patients undergoing elective surgery that lasted more than 4 h (240 min) - American Society of Anaesthesiologists (ASA) from 4 to 5

The following baseline demographic variables were collected: age, gender, height, weight and body mass index (BMI). Operative variables collected included the type and duration of the operation. The anaesthesiologists were blinded to the patients’ participation in the study, and the NMBDs were supervised according to the standards of patient care in PACU and whether these patients required oxygen following their extubation in the operating theatre.

On arrival at the PACU, the type and dose of neuromuscular blockade drugs (NMBDs) and reversal drugs and the duration of surgery are recorded by qualified nurses in PACU. They also measured the following postoperative outcomes: peripheral oxygen saturation (SpO_2_), respiratory rate (RR) and TOF ratio during the first 30 min of arrival into the PACU in which all of them were obtained by the researcher. The research assistant obtained TOF ratio via acceleromyography (quantitative neuromuscular monitoring) at the adductor pollicis of the thumb (TOF-Scan™, IdMed, Marseille, France). When the patients experienced any of CREs based on the modified Murphy’s criteria within the first 30 min in admission to the PACU, the nurses have directly informed the researcher about these changes in the patients’ condition as shown in Table 2.

**Table 2:** Types of critical respiratory events (modified Murphy’s criteria)

1. Upper airway obstruction: if the patient required an intervention such as: o Oral airway o Jaw thrust o Nasal airway 2. Mild-moderate hypoxaemia (SpO_2_ of 93–90%) 3. Severe hypoxaemia (SpO_2_ < 90%) 4. Signs of respiratory distress or impending ventilation failure: o Accessory muscle use o Tracheal tug o Respiratory rate > 20 breaths per minute 5. Patient could not breathe deeply whilst asked for by the PACU nursing staff 6. Patient complaining of upper airway muscle weakness or symptoms of respiratory: o Difficulty swallowing: ask the patient to swallow a sip of water o Difficulty speaking o Difficulty breathing 7. Patient requiring re-intubation in the PACU 8. Clinical evidence or suspicion of pulmonary aspiration after tracheal extubation (gastric contents observed in the oropharynx and hypoxaemia)	

### Statistical analysis

All statistical analysis for the current study was performed by using the SPSS (version 23.0). Categorical data were presented as percentages or frequencies, and the chi-square test was used for comparison. For non-normally distributed data, the Mann-Whitney *U* test, median and interquartile range (IQR) were used, whilst the mean, standard deviation (SD) and independent-samples *T* tests were used if normally distributed. A *p* value of less than 0.05 was considered for the statistically significant level.

## Results

**Fig. 1:**
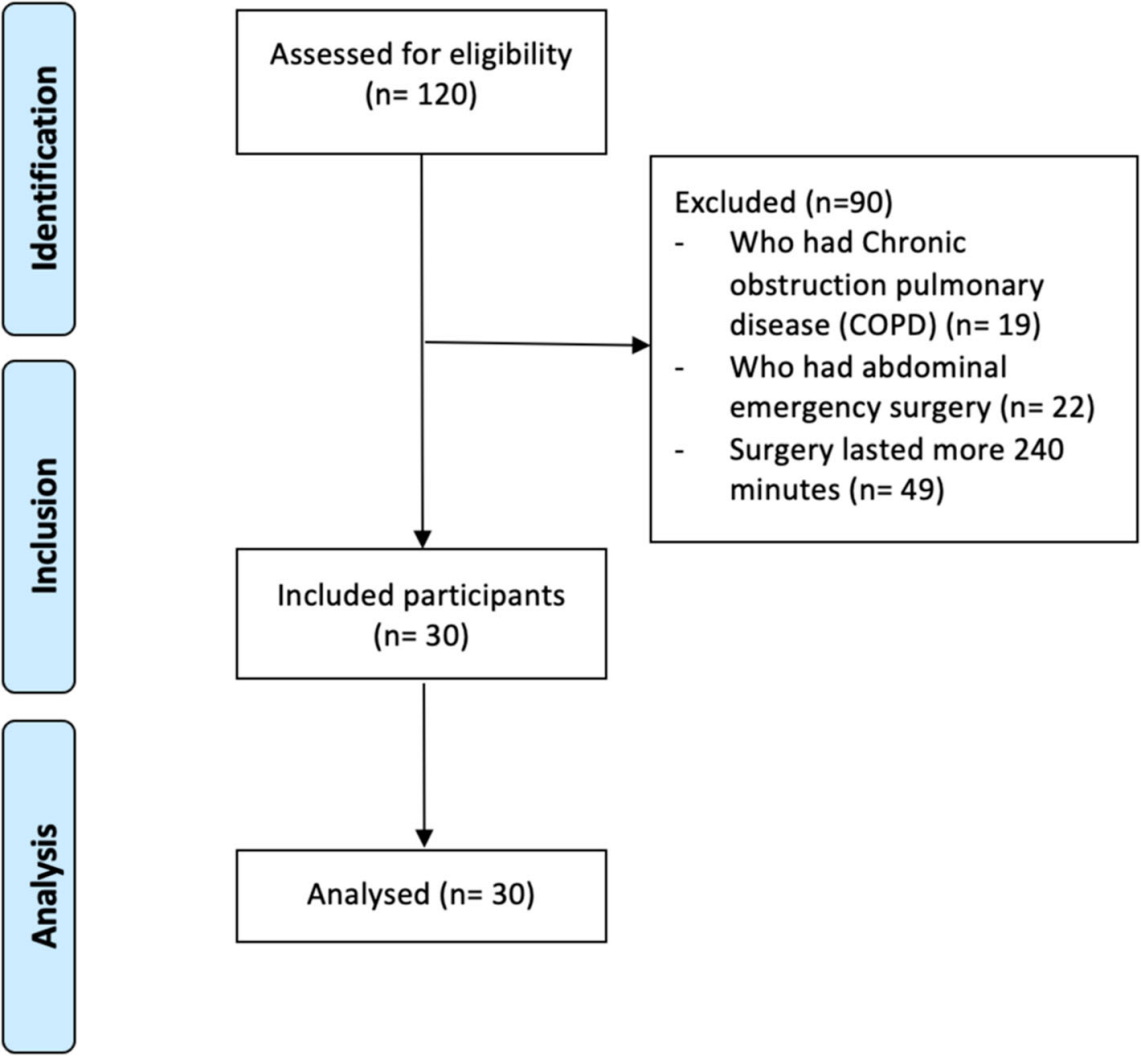
Flow diagram of participants’ enrolment

A total of 120 patients were screened for their eligibility based on the inclusion and exclusion criteria at a pre-anaesthesia clinic over a period of 4 weeks (Table 1). Nineteen patients with chronic obstructive pulmonary disease (COPD), 22 with abdominal emergency surgery and 49 who undergone elective surgery that lasted more than 4 h (240 min) were excluded. A total of 30 patients, consisting of 17 males and 13 females, were recruited for analysis (Fig. 1).

Demographic data of the population, surgical and anaesthesia details are summarised in Table 3. The median age of participants was 35 years. The majority of these participants were male (56.7%), whilst female patients represented 43.3% of the sample. Based on Fortier et al. (2015) and Murphy et al. (2015), patients’ age was categorized into two age groups: younger patients (age 18 to 50 years) and elderly patients (age ≥ 51 years). More than half of the studied patients had American Society of Anaesthesiologists Physical Status (ASA-PS) class II (56.7%), followed by ASA-PS class I (33.3%). The majority of the cohort was undergoing general surgeries (43.3%) and ENT surgeries (33.3%), followed by urology surgeries (13.3%). The most frequently used NMBD during the current study was rocuronium with an initial dose of 50 mg (86.7%), whilst the most frequently used reversal drug was neostigmine (76.7%). The mean BMI among the participants was 29.45 ± 5.56 kg/m^2^.

**Table 3:** Demographic data

Variables	Findings (*n* = 30)
Age (years), median (IQR)	35 (27–53)
Gender, *n* (%)	
Male	17(56.7%)
Female	13(43.3%)
ASA-PS class, *n* (%)	
I	10 (33.3%)
II	17 (56.7%)
III	3 (10%)
BMI (kg/m_2_), mean ± SD	29.45 ± 5.56
Type of surgery, *n* (%)	
General	13 (43.3%)
Urology	4 (13.3%)
ENT	10 (33.3%)
Plastic	2 (6.7%)
OB/GYN	1 (3.3%)
NMBD used, *n* (%)	
Succinylcholine	1 (3.3%)
Rocuronium	26 (86.7%)
Cisatracurium	3 (10%)
Reversal used, *n* (%)	
None	7 (23.3%)
Neostigmine	23 (76.7%)

*n (%)* number (percentage), *IQR* interquartile range, ± *SD* ± standard deviation, *ASA*-*PS class* American Society of Anaesthesiologists Physical Status class, *BMI* body mass index, *kg*/*m*^*2*^ kilogramme per metre square, *ENT* ear, nose and throat, *OB*/*GYN* obstetrics and gynaecology, *NMBD* neuromuscular blocking drug

**Fig. 2:**
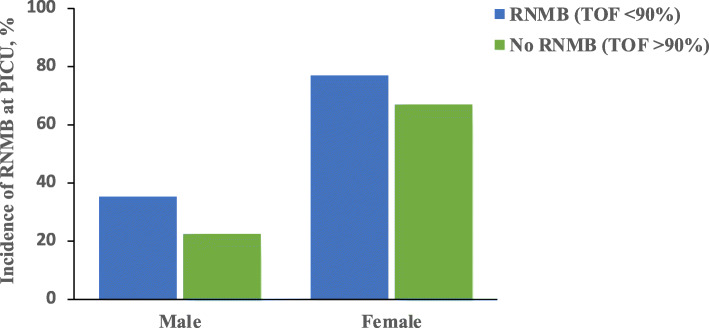
The incidence of RNMB among the sample based on their gender

The incidence of RNMB (TOF ratio < 0.90) was 53.3% (16/30). In comparing the preoperative variables among patients who had RNMB on PACU admission, the chi-square test clearly showed that there were no significant differences between the incidence of RNMB according to the age of patients (18–50 vs. ≥ 51 years) or ASA-PS class, with *p* = 0.0544 and *p* = 0.103, respectively. The same test demonstrated that the incidence of the RNMB was significantly higher in the female gender (76.9% vs. 35.3%, *p* = 0.033) (Fig. 2), in patients who had not had neuromuscular monitoring before extubation (66.7% vs. 22.2%, *p* = 0.046), and in shorter duration of surgery (81.8% vs. 21.4%, *p* = 0.001). The independent-samples *T* tests demonstrated no significant differences in RNMB in relation to BMI (*p* = 0.294). In comparing patients who had RNMB upon arrival in the PACU with those who did not, the Mann-Whitney *U* test demonstrated a significant relationship between the incidence of RNMB and both oxygen desaturation and respiratory rate, at *p* = 0.034 and *p* = 0.025, respectively (Table 4).

**Table 4:** Patient characteristics and the incidence of RNMB

Variables	RNMB (TOF ratio < 0.90), *n* = 16	No RNMB (TOF ratio ≥ 0.90), *n* = 14	*P* value
Age, *n* (%)			
18–50 vs. ≥ 51 years	11 (50%) vs. 5 (62.5%)	11 (50%) vs. 3 (37.5%)	0.0544
Gender, *n* (%)			
Male/female	6 (35.3%)/10 (76.9%)	11 (64.7%)/ 3 (23.1%)	0.033
ASA-PS class, *n* (%)			
I	7 (43.8%)	3 (21.4%)	
II	9 (56.3%)	8 (57.1%)	0.103
III	0 (0.0%)	3 (21.4%)	
BMI (kg/m^2^), mean ± SD	30.82 ± 5.29	28.53 ± 6.41	0.294
Neuromuscular monitoring before extubation, *n* (%)			
Yes/no	2 (22.2%)/14 (66.7%)	7 (77.8%)/7 (33.3%)	0.046
Duration of the surgery, < 90 min vs. ≥ 90 min	13 (81.8%) vs. 3 (21.4%)	3 (18.8%) vs. 11 (78.6%)	0.001
SpO_2_, upon arrival in the PACU, median (IQR)	91 (90–93)	96 (91–100)	0.034
RR (breaths/min), upon arrival in the PACU, median (IQR)	22 (19–25)	20 (16–21)	0.025

*n (%)* number (percentage), *IQR* interquartile range, ± *SD* ± standard deviation, *RNMB* residual neuromuscular blockade, *ASA*-*PS class* American Society of Anaesthesiologists Physical Status class, *BMI* body mass index, *kg*/*m*^*2*^ kilogramme per metre square, *SpO*_*2*_ oxygen saturation, *RR* respiratory rate, *min* minutes, *TOF* train-of-four, *PACU* post-anaesthesia care unit

Overall, 66.7% of patients who underwent elective surgeries under general anaesthesia experienced postoperative CREs, compared with 33.3% who did not (Table 5). The Mann-Whitney *U* test revealed that there was no significant difference in postoperative CREs in the PACU between age groups and groups with long vs. short surgery times. The median of the TOF ratio upon arrival in the PACU was 0.88 in patients with postoperative CREs. The chi-square test demonstrated that RNMB (TOF ratio < 0.90) was more frequent among those participants with postoperative CREs (75% vs. 10%, *p* = 0.001). The Mann-Whitney *U* test demonstrated no significant association between the patients’ temperature and the incidence of CREs. The independent-samples *T* tests showed a significant difference in postoperative CREs between the two BMI groups (31.23 ± 6.1 vs. 26.78 ± 3.94, *p* = 0.047).

**Table 5:** Comparison between the variables with the presence or absence of CREs upon arrival in the PACU

Variables	CRE (*n* = 20)	No CREs (*n* = 10)	*P* value
Age (years), median (IQR)	35 (27–51)	35 (30–53)	0.62
BMI (kg/m^2^), mean ± SD	31.23 ± 6.17	26.78 ± 3.94	0.047
Duration of surgery (min), median (IQR)	82 (75–161)	106 (86–158)	0.403
Temperature (°C), median (IQR)	36.50 (36.40–36.80)	36.65 (36.40–36.90)	0.689
TOF ratio upon arrival in the PACU, median (IQR)	0.88 (0.72–0.89)	0.96 (0.91–1.00)	0.001
Incidence of RNMB (TOF ratio < 0.90), *n* (%)	15 (75%)	1(10%)	0.001

*n (%)* number (percentage), *IQR* interquartile range, ± *SD* ± standard deviation, *CREs* critical respiratory events, *BMI* body mass index, *kg*/*m*^*2*^ kilogramme per metre square, *min* minutes, *TOF* train-of-four, *PACU* post-anaesthesia care unit, *RNMB* residual neuromuscular blockade

During the present study, the incidence of critical respiratory events in the PACU was defined according to the described criteria by Murphy et al. (2008a). Based on these criteria, twenty patients experienced postoperative CREs during the first 30 min in the PACU in this current study. The one or two signs of CREs commonly observed in the sample of this study with respiratory distress (50%), mild to moderate hypoxaemia (30%) and upper airway obstruction (16.7%). The other CREs recorded were severe hypoxaemia (13.3), upper airway weakness (10%) and inability to breathe deeply (6.7%) as in Table 6. None of the patients had pulmonary respiratory disease, and none of them required re-intubation in the PACU stay.

**Table 6:** Incidence of CREs in the PACU

Variable	Incidence
Upper airway obstruction	5 (16.7%)
Mild-moderate hypoxaemia	9 (30%)
Severe hypoxaemia	4 (13.3%)
Signs of respiratory distress	15 (50%)
Inability to breathe deeply	2 (6.7%)
Upper airway weakness	3 (10 %)

## Discussion

The aim of this pilot study was to understand the baseline incidence of RNMB and postoperative CREs, the feasibility of measuring TOF during the PACU stay in a tertiary hospital in Saudi Arabia. The incidence of RNMB in our cohort was 53.3%, and postoperative CREs were observed among 66.7% of our cohort. The findings show that there was a statistically significant association between the incidence of RNMB and postoperative CREs. The finding of this study is consistent with the POPULAR study by Kirmeier et al. (2019), a recently published multicentre prospective observational study that examined 22803 patients who had undergone general anaesthesia. In this study, researchers have concluded that using the NMBDs was associated with an increased incidence of postoperative pulmonary complications (7.6%, 1658/ 21694; odd ratio 1.86, 95% CI 1.53–2.26).

Based on the patients’ demographic data and preoperative variables, which are presented in Table 4, the results of the current study do not show that female patients were significantly more likely to experience RNMB in the PACU than males (*p* = 0.033) as shown in Table 4. These findings are consistent with other studies conducted by Aytac et al. (2016), Pietraszewski and Gaszyński (2013) and Kaan et al. (2012). This could be that women are more sensitive to rocuronium, the onset time of rocuronium is shorter in women and its effective period is prolonged (Xue et al. 1997 and Adamus et al. 2008). They also added that the variability of sensitivity and action duration of NMBDs between women and men is believed to be associated with physiological differences in body structures. Whilst Aytac et al. (2016) also found that female patients undergoing anaesthesia of short duration are more likely to have RNMB in the PACU. Therefore, both Xue et al. (1997) and Adamus et al. (2008) suggest that the dose of rocuronium routinely used in females could be reduced.

Other significant findings in the current studies that the incidence of RNMB is significantly more frequent among patients for whom neuromuscular monitoring (acceleromyography) has not been used prior to extubation (*p* = 0.046). Several observational studies conducted in different countries (Fuchs-Buder et al. 2003; Grayling and Sweeney 2007; Sorgenfrei et al. 2005) have reported similar to this, but they cannot be directly compared because they used different techniques for neuromuscular monitoring. And this was also supported by Murphy et al. (2008b) who found that RNMB was more common in the conventional monitoring group than that in the quantitative monitoring group (*p* < 0.001).

The findings of this study suggest that there was a significant difference in the incidence of RNMB in relation to the duration of surgery whether short or long (< 90 min compared with ≥ 90 min; *p* = 0.001). Stewart et al. (2016) also showed agreement with the findings as the RNMB was significantly more likely to be associated with shorter operations (*p* = 0.001). The agreement among the conclusions of these two studies could be referred to the fact that sufficient time is required for neostigmine to provide effective reversal, or as Miller (2011) for spontaneous recovery from neuromuscular blockade to a TOF ratio ≥ 0.90, which may not be allowed during shorter surgery times.

Despite advances in the neuromuscular monitoring methods and pharmacological agents used in anaesthesia practice, the findings of this study showed that postoperative CREs were significantly associated with RNMB (*p* = 0.001), which was consistent with the previous studies (Grosse-Sundrup et al. 2012; Murphy et al. 2008a; Murphy et al. 2008b; Norton et al. 2013; Sauer et al. 2011) as shown in Table 5. Murphy et al. (2008b) in their study have examined the effectiveness of quantitative (acceleromyography) and qualitative neuromuscular monitoring in reducing the incidence of RNMB after tracheal extubation. They found that RNMB (TOF ratio < 0.90) was more frequent in the conventional group (30%) compared with the acceleromyography group (4.5%; *p* < 0.001). In addition, the authors revealed that mild hypoxaemia (SpO_2_ 90–93%) was significantly more frequent in the conventional group (43.3% vs. 6.7%), and severe hypoxaemia (SpO_2_ < 90%) was also more common in the conventional group during the first 30 min of their PACU stay (21.1%; all *p* < 0.001). The result of Murphy et al. (2008b) suggested that RNMB is significantly associated with hypoxaemia, which was similar to the findings of the current study.

Postoperative CREs occurred in twenty patients (66.7%), and there were significantly more of these CREs among patients with RNMB (*p* = 0.001). Xará et al. (2015) examined 340 patients and found that CREs happened in 67 of them (19.7%), and CREs were more frequent in patients with RNMB (*p* < .001). Moreover, among the 599 patients studied by Stewart et al. (2016), 97 participants experienced one or more CREs in the PACU, and these CREs were more frequent in patients with RNMB (*p* = 0.033). In addition, Stewart et al. (2016) observed some CREs that were not observed by Xará et al. (2015) or by the current study, such as pulmonary aspiration after tracheal extubation or the need for assisted ventilation. However, the differences in findings among these studies could be impacted by either the sample size or the type of patients recruited during the studies.

There were no significant differences between the age of patients and the incidence of postoperative CREs (*p* = 0.62) in the current study. However, Cedborg et al. (2014) found that there was an intense negative effect on airway integrity in elderly patients with increased prevalence of pharyngeal dysfunction: from 37% in patients without RNMB to 71% during the presence of RNMB. Pedersen (1994) examined the risk factors associated with postoperative complications following general anaesthesia and found that postoperative pulmonary complications were associated with elderly patients. Therefore, the differences in the complications among the previous studies and the current study might be impacted by the age of their samples included (Sieber and Barnett 2011).

The results of this study seem to suggest a significant association between BMI and postoperative CREs during the early PACU stay. BMI (31.23 ± 6.1 kg/m^2^) was significantly greater (*p* = 0.047) among patients who experienced CREs compared to those who did not (26.78 ± 3.94 kg/m^2^). According to the World Health Organization (2016), subjects with BMI equal to or greater than 30 kg/m^2^ are considered obese. Obesity has physiological effects on the respiratory system’s compliance and lung volumes and that BMI was significantly associated with severe hypoxaemia (SpO_2_ < 90%) during the PACU stay (*p* = 0.01) (Hodgson et al. 2015; Cammu et al. (2012).

The findings reported here can improve the current knowledge and clinical practice of perioperative management, specifically the management of NMBDs. The first step for improving NMBDs’ management and postoperative outcomes is basically through following the evidence-based medical management of NMBDs, which can help to reduce complications initiated by unsuitable perioperative management. Therefore, based on the current literature and AAGBI guidelines (2016), the researcher recommends that neuromuscular monitoring in the operating theatre before extubation is required for all patients who have received NMBDs. Quantitative neuromuscular monitoring (acceleromyography) is essential to accurately assess the TOF ratio, which can help to diminish the incidence of RNMB. This will also help to prevent the postoperative complications associated with RNMB and that will reduce the incidence of postoperative critical respiratory events.

The current pilot study has some limitations that need to be mentioned. The convenience sampling method, time frame and sample size were applicable and appropriate for this study. The researcher was limited by resources and time boundaries; hence, the sample employed was small, which might reduce the external validity of the findings of the current study (LoBiondo-Wood and Haber 2014). However, the lack of generalizability and sampling bias are the main disadvantages of this sampling method (Polit 2014). The study was carried out only on elective surgical patients in a single tertiary hospital, and therefore, the results might be difficult to generalise to other surgical populations or to different settings (Greenhalgh 2014). Moreover, the present study was a prospective, observational study: no interventions were made in anaesthetic practice during the pre-operative, intra-operative or post-operative period, and all treatments were applied and managed according to the standards of the clinician who was in charge of each patient that day.

Further research into the association between the incidence of RNMB and postoperative CREs following general anaesthesia should address the limitations of this pilot study. With a CRE rate of 66%, this pilot study certainly demonstrates the need for further multicentre, randomised trials in Saudi Arabia to provide better findings and more conclusive results. Enrolment of large samples is necessary for the appropriate detection of associations with the incidence of RNMB and postoperative CREs.

## Conclusion

This is the first study conducted in Saudi Arabia that has investigated the baseline incidence of RNMB and postoperative CREs among patients undergoing elective surgery under general anaesthesia. This study observed evidence that the majority of patients with RNMB (TOF ratio < 0.90) experienced CREs in the PACU. In this study, the analysis demonstrated that high BMI (31.23 ± 6.1 kg/m^2^) can also contribute to CREs during the early PACU stay. Through further descriptive analysis of the sample characteristics, it was shown that female gender, shorter duration of surgery and the absence of intraoperative quantitative neuromuscular monitoring were the variables that were most likely to lead to the occurrence of RNMB (TOF ratio < 0.90).

In agreement with previous researches, the current study confirms the continued high incidence of RNMB upon PACU arrival during regular clinical practice, despite the use of neostigmine and qualitative TOF monitoring. Routine quantitative neuromuscular monitoring is recommended to enhance patient safety. Therefore, these results should provoke a re-evaluation of current practice and techniques used for the monitoring and reversal of NMBDs in anaesthesia practice in Saudi Arabia. The limitations of this research, as highlighted above, along with the suggested recommendations for future practice, will provide further evidence to supplement the current research and the extant literature, which is essential in improving the quality of anaesthesia practice.

## Data Availability

Available

## Abbreviations

PACU: Post-anaesthesia care units
NMBDs: Neuromuscular blocking drugs
NMBD: Neuromuscular blocking drug
RNMB: Residual neuromuscular blockade
TOF: Train-of-four
CREs: Critical respiratory events
BMI: Body mass index
ASA-PS: American Society of Anaesthesiologists Physical Status
SpO_2_: Oxygen saturation
RR: Respiratory rate
